# Supplementary material for: GTSF1 accelerates target RNA cleavage by PIWI-clade Argonaute proteins
Source: Nature. 2022 Jun 30;608(7923):618–25. doi: 10.1038/s41586-022-05009-0 (PMC9385479; doi:10.1038/s41586-022-05009-0)
Supplement: Supplementary file 1 — Reporting Summary [file 41586_2022_5009_MOESM1_ESM.pdf]

nature portfolio

Corresponding author(s): Phillip D. Zamore

Last updated by author(s): May 18, 2022

Reporting Summary

Nature Portfolio wishes to improve the reproducibility of the work that we publish. This form provides structure for consistency and transparency in reporting. For further information on Nature Portfolio policies, see our Editorial Policies and the Editorial Policy Checklist.

Statistics

For all statistical analyses, confirm that the following items are present in the figure legend, table legend, main text, or Methods section.

n/a	Confirmed	
☐	☒	The exact sample size (*n*) for each experimental group/condition, given as a discrete number and unit of measurement
☒	☐	A statement on whether measurements were taken from distinct samples or whether the same sample was measured repeatedly
☐	☒	The statistical test(s) used AND whether they are one- or two-sided<br>*Only common tests should be described solely by name; describe more complex techniques in the Methods section.*
☒	☐	A description of all covariates tested
☒	☐	A description of any assumptions or corrections, such as tests of normality and adjustment for multiple comparisons
☐	☒	A full description of the statistical parameters including central tendency (e.g. means) or other basic estimates (e.g. regression coefficient) AND variation (e.g. standard deviation) or associated estimates of uncertainty (e.g. confidence intervals)
☐	☒	For null hypothesis testing, the test statistic (e.g. *F*, *t*, *r*) with confidence intervals, effect sizes, degrees of freedom and *P* value noted<br>*Give P values as exact values whenever suitable.*
☒	☐	For Bayesian analysis, information on the choice of priors and Markov chain Monte Carlo settings
☒	☐	For hierarchical and complex designs, identification of the appropriate level for tests and full reporting of outcomes
☒	☐	Estimates of effect sizes (e.g. Cohen's *d*, Pearson's *r*), indicating how they were calculated

Our web collection on statistics for biologists contains articles on many of the points above.

Software and code

Policy information about availability of computer code

Data collection	Typhoon FLA 7000, NanoAcquity UPLC, Orbitrap Fusion Lumos tribrid, Li-CoR Odyssey Clx, Image Studio
Data analysis	Igor Pro 8, Microsoft Excel 16.59, Bowtie 2.2.5, STAR 2.3, SAMtools 1.8, HTSeq 0.9.1, Proteome Discoverer 2.1.1.21, Mascot 2.6.2, Scaffold 4.8.9, Image Studio Lite (5.2.5), MacVector 18.0.1, Clustal Omega 1.2.4, RAxML (1.0.0)

For manuscripts utilizing custom algorithms or software that are central to the research but not yet described in published literature, software must be made available to editors and reviewers. We strongly encourage code deposition in a community repository (e.g. GitHub). See the Nature Portfolio guidelines for submitting code & software for further information.

Data

Policy information about availability of data

All manuscripts must include a data availability statement. This statement should provide the following information, where applicable:

- Accession codes, unique identifiers, or web links for publicly available datasets
- A description of any restrictions on data availability
- For clinical datasets or third party data, please ensure that the statement adheres to our policy

All data are available from the authors upon request.
Swiss-Prot human database (download 04/09/2019; https://www.uniprot.org/)

Field-specific reporting

Please select the one below that is the best fit for your research. If you are not sure, read the appropriate sections before making your selection.

☒ Life sciences ☐ Behavioural & social sciences ☐ Ecological, evolutionary & environmental sciences

For a reference copy of the document with all sections, see nature.com/documents/nr-reporting-summary-flat.pdf

Life sciences study design

All studies must disclose on these points even when the disclosure is negative.

Sample size	No statistical method was used to determine sample size. Three replicates is standard for biochemical assays.
Data exclusions	No data were excluded from the analysis.
Replication	The number of times each experiment was performed is specified in the "Statistics and Reproducibility" section of the methods. All attempts at replication were successful.
Randomization	Randomization is not relevant to this study. Biochemical experiments are rarely randomized.
Blinding	Blinding was not performed during data acquisition or analysis. Biochemical experiments are rarely blinded

Reporting for specific materials, systems and methods

We require information from authors about some types of materials, experimental systems and methods used in many studies. Here, indicate whether each material, system or method listed is relevant to your study. If you are not sure if a list item applies to your research, read the appropriate section before selecting a response.

Materials & experimental systems

n/a	Involved in the study	
☐	☒	Antibodies
☐	☒	Eukaryotic cell lines
☒	☐	Palaeontology and archaeology
☐	☒	Animals and other organisms
☒	☐	Human research participants
☒	☐	Clinical data
☒	☐	Dual use research of concern

Methods

n/a	Involved in the study	
☒	☐	ChIP-seq
☐	☒	Flow cytometry
☒	☐	MRI-based neuroimaging

Antibodies

Antibodies used	Anti-FLAG antibody (M2, Sigma F1804); anti-BmAgo3 (Created by Izumi in: Izumi et al., 2020, Nature 578, 311-316 and used by Izumi in this manuscript); alpha-Tubulin antibody #2144 (https://www.cellsignal.com/)
Validation	Anti-FLAG antibody (https://www.sigmaaldrich.com/content/dam/sigma-aldrich/docs/Sigma/Bulletin/f1804bul.pdf); anti-BmAgo3 was produced and validated in a previous study (Izumi et al., 2020, Nature 578, 311-316); anti-alpha-Tubulin antibody (https://www.cellsignal.com/)

Eukaryotic cell lines

Policy information about cell lines

Cell line source(s)	HEK 293T (lab stock; commercially available from ATCC CRL-3216) and BmN4 cell line (provided by Dr. Kusakabe, Kyushu University and available for purchase from Riken Cell Bank- https://cellbank.brc.riken.jp/cell_bank/CellInfo/?cellNo=RCB2126&lang=En)
Authentication	The cells were not authenticated; cells were used only to produce recombinant proteins
Mycoplasma contamination	Not tested.
Commonly misidentified lines (See ICLAC register)	No commonly misidentified lines were used in the study.

Animals and other organisms

Policy information about studies involving animals; ARRIVE guidelines recommended for reporting animal research

Laboratory animals	C57BL/6 mice: JAX#000664; adult male<br>FLAG-Gtsf1KI mice: generated in the C57BL/6J background in this study; adult male.<br>Animals were housed in an AALAC-accredited barrier facility with controlled temperature (22°± 2°), relative humidity (40% ± 15%), and a 12-hour dark/light cycle.<br><br>All animals used in experiments were two to six months old.
Wild animals	No wild animals were used in this study.
Field-collected samples	No field-collected samples were used in this study.
Ethics oversight	(1) PI on IACUC protocol: Phillip D. Zamore<br>(2) Name of IACUC: UMass Chan Medical School Institutional Animal Care and Use Committee<br>(3) IACUC Docket: A2222-17, "Investigation of mechanisms of small RNA function in vivo"<br>(4) Mice were maintained and used according to the guidelines of the Institutional Animal Care and Use Committee of the University of Massachusetts Chan Medical School (A201900331).

Note that full information on the approval of the study protocol must also be provided in the manuscript.

Flow Cytometry

Plots

Confirm that:

- [ ] The axis labels state the marker and fluorochrome used (e.g. CD4-FITC).
- [ ] The axis scales are clearly visible. Include numbers along axes only for bottom left plot of group (a 'group' is an analysis of identical markers).
- [ ] All plots are contour plots with outliers or pseudocolor plots.
- [ ] A numerical value for number of cells or percentage (with statistics) is provided.

Methodology

Sample preparation	*Describe the sample preparation, detailing the biological source of the cells and any tissue processing steps used.*
Instrument	*Identify the instrument used for data collection, specifying make and model number.*
Software	*Describe the software used to collect and analyze the flow cytometry data. For custom code that has been deposited into a community repository, provide accession details.*
Cell population abundance	*Describe the abundance of the relevant cell populations within post-sort fractions, providing details on the purity of the samples and how it was determined.*
Gating strategy	*Describe the gating strategy used for all relevant experiments, specifying the preliminary FSC/SSC gates of the starting cell population, indicating where boundaries between "positive" and "negative" staining cell populations are defined.*

- [ ] Tick this box to confirm that a figure exemplifying the gating strategy is provided in the Supplementary Information.
