# Supplementary material for: GTSF1 accelerates target RNA cleavage by PIWI-clade Argonaute proteins
Source: Nature. 2022 Jun 30;608(7923):618–25. doi: 10.1038/s41586-022-05009-0 (PMC9385479; doi:10.1038/s41586-022-05009-0)
Supplement: Supplementary file 2 — Uncropped source gel images. [file 41586_2022_5009_MOESM2_ESM.pdf]

Arif et al., Supplementary Data Fig. 1.

Extended Data Fig. 1b

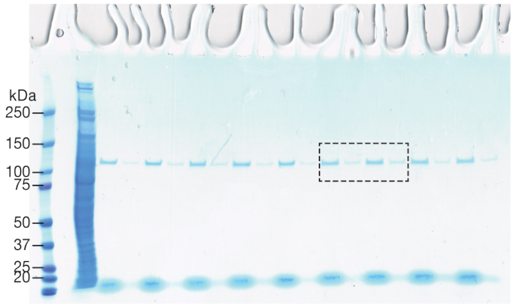

Extended Data Fig. 1c

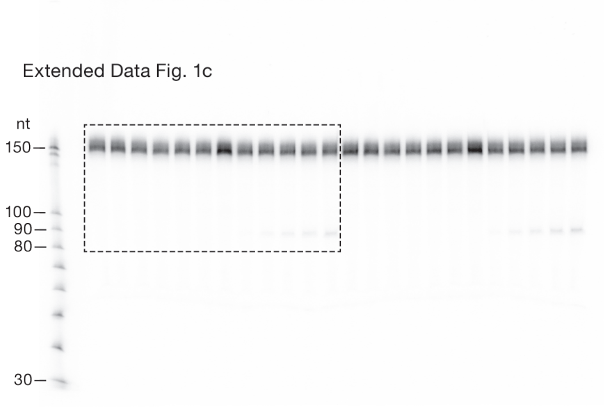

Extended Data Fig. 1d

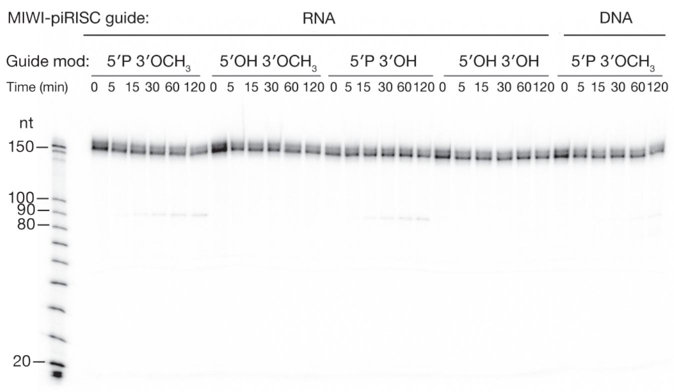

Extended Data Fig. 1e

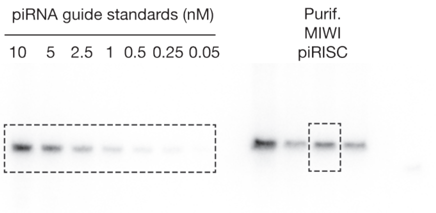

Extended Data Fig. 1f

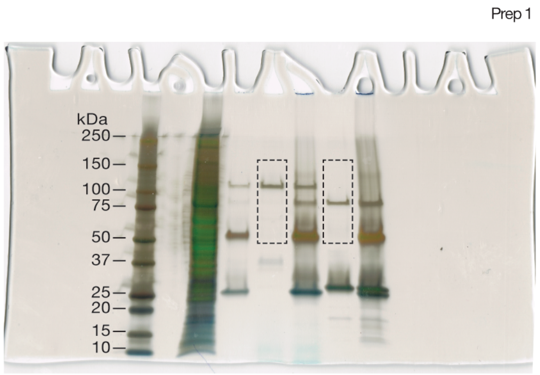

Extended Data Fig. 1g

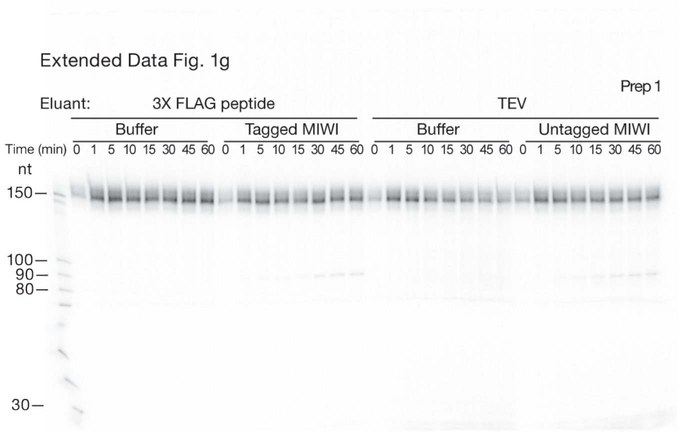

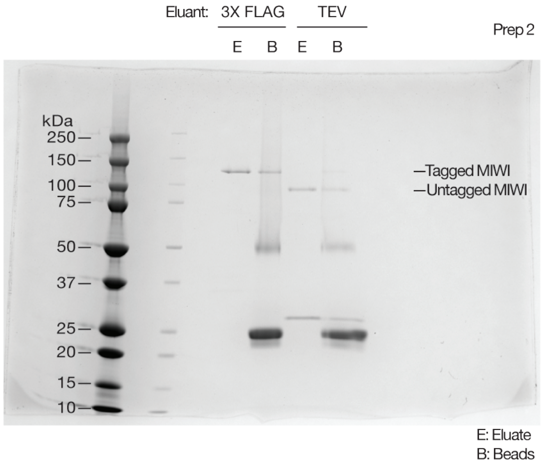

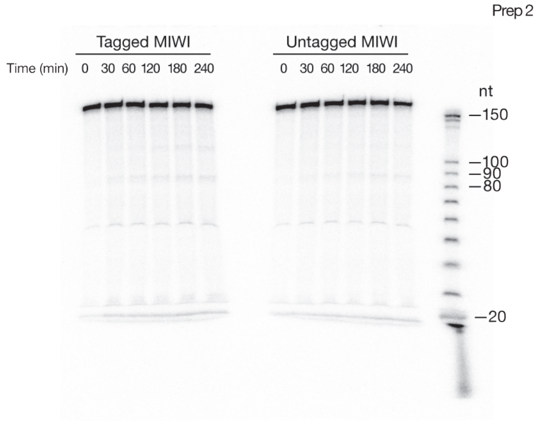

Figure 1b (top)

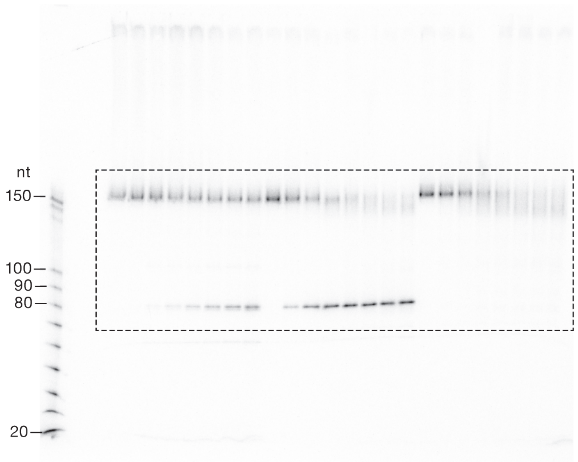

Figure 1b (bottom) and Figure 3a, 3b (5 nM target)

Trial 1

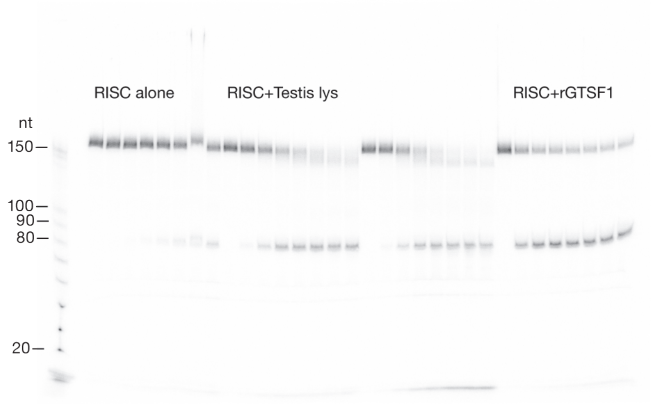

Trial 2

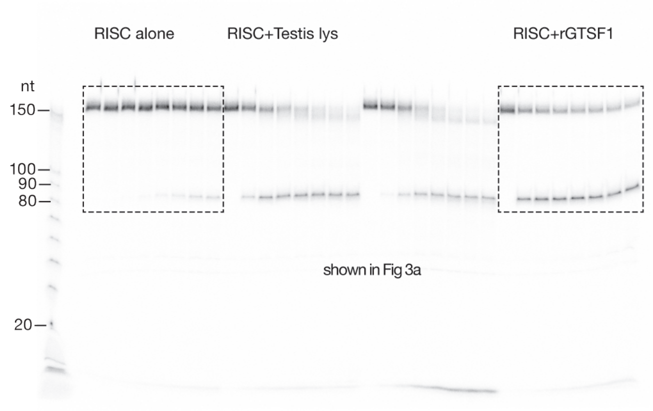

Figure 1c

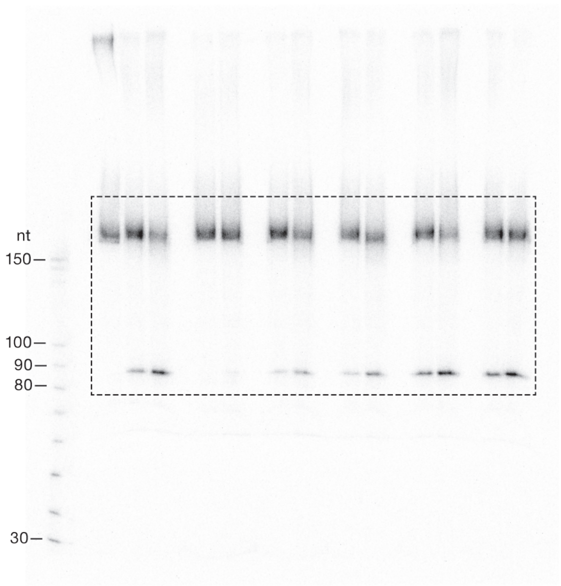

Trial 3

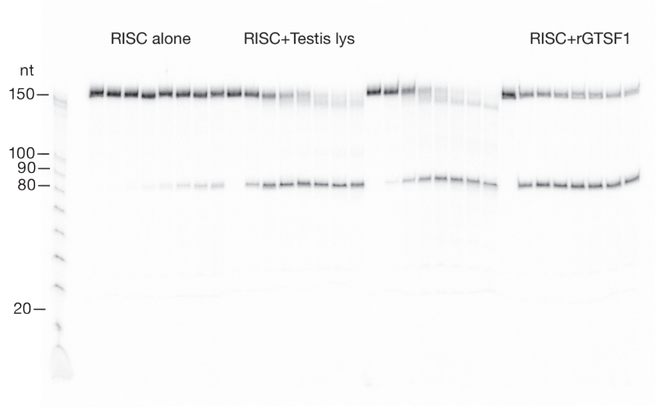

Extended Data Fig. 2a

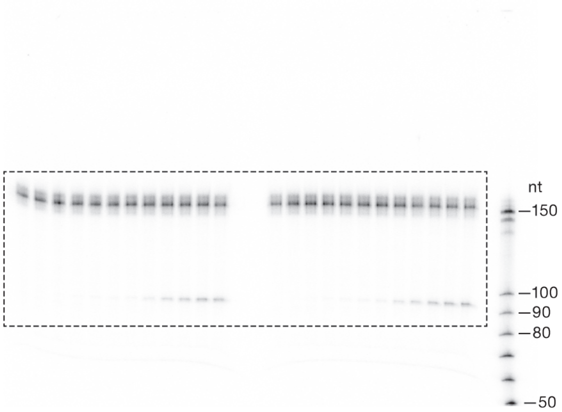

Extended Data Fig. 2b

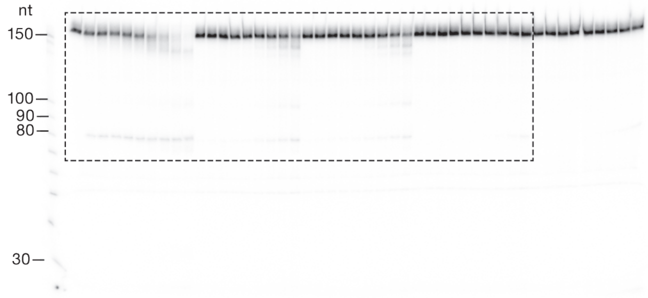

Arif et al., Supplementary Data Fig. 1.

Extended Data Fig. 3a

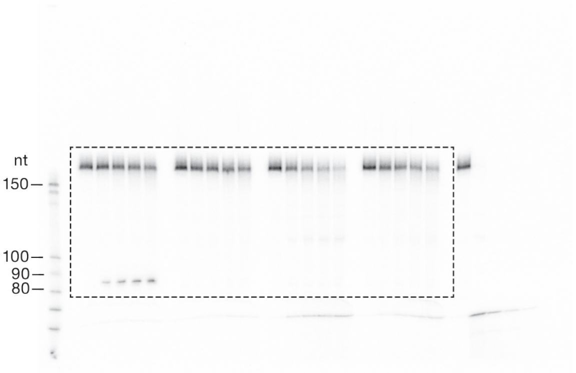

Extended Data Fig. 3b

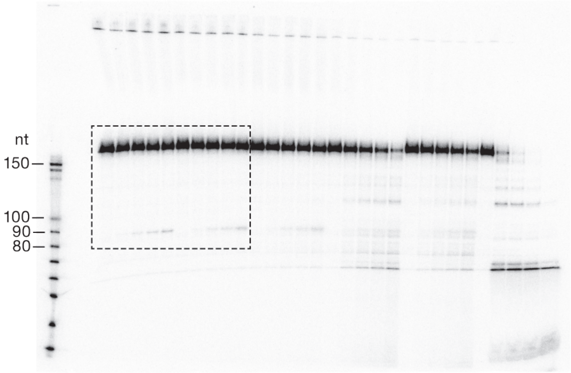

Extended Data Fig. 3c

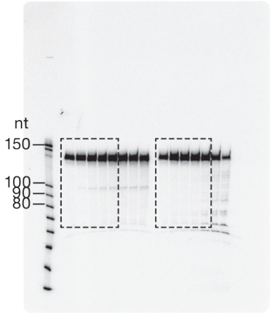

Extended Data Fig. 3d

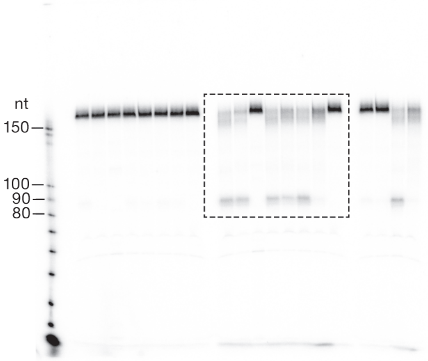

Extended Data Fig. 3f

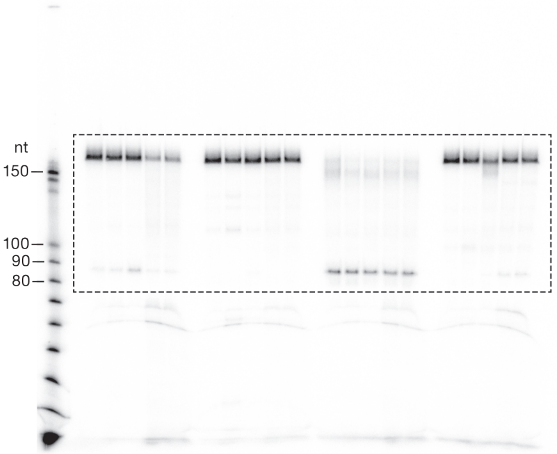

Extended Data Fig. 3g

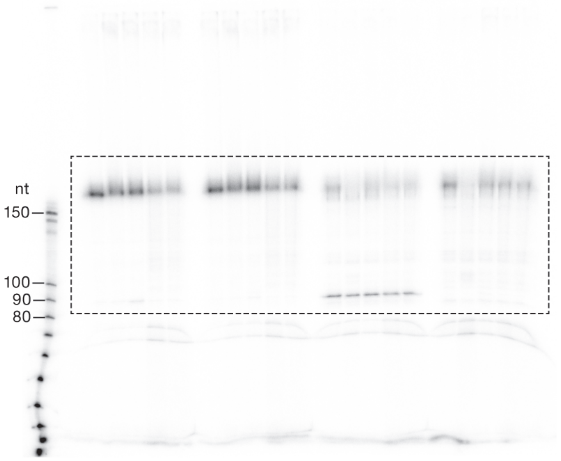

Extended Data Fig. 3h

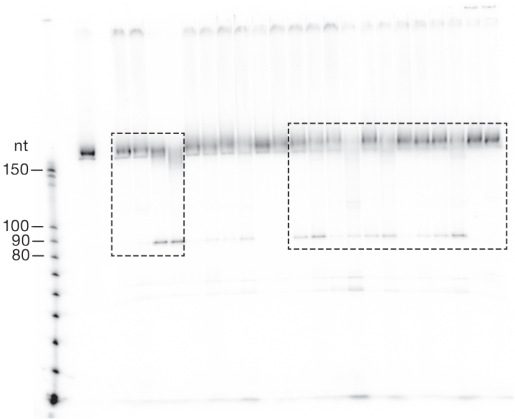

Arif et al., Supplementary Data Fig. 1.

Figure 2b

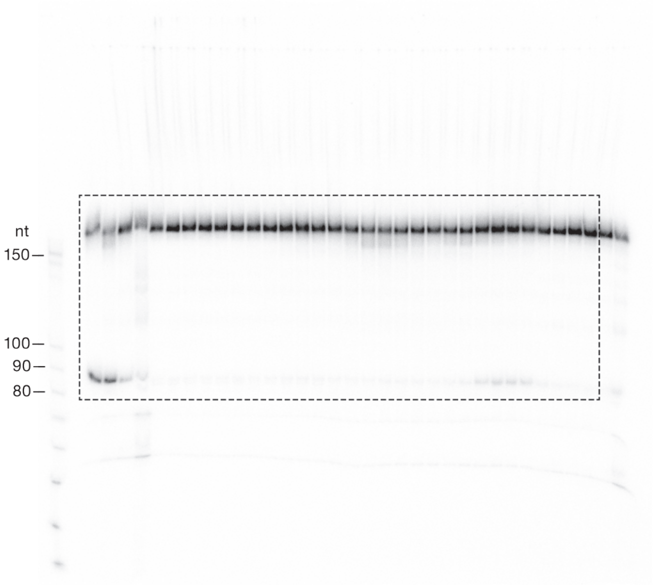

Figure 2e

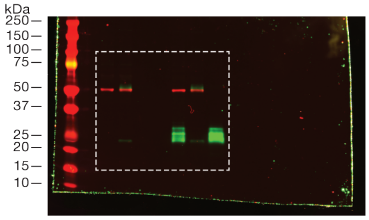

Extended Data Figure 4b

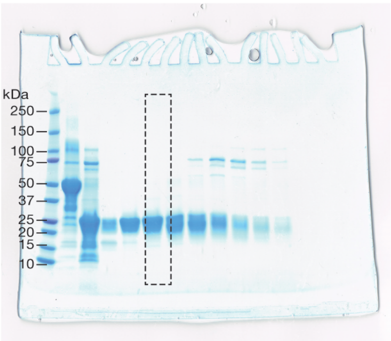

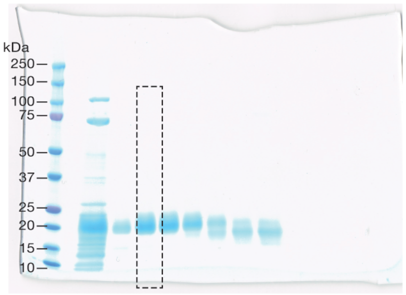

Figure 2f

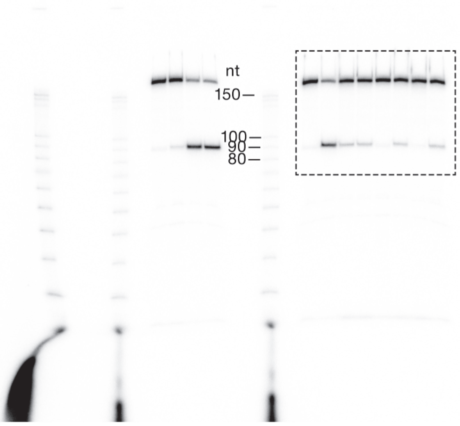

Extended Data Figure 4b (contd)

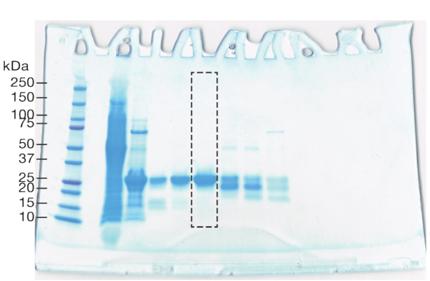

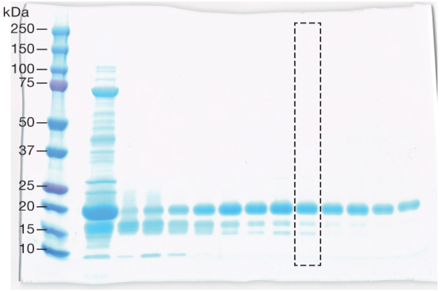

Extended Data Figure 4b (contd)

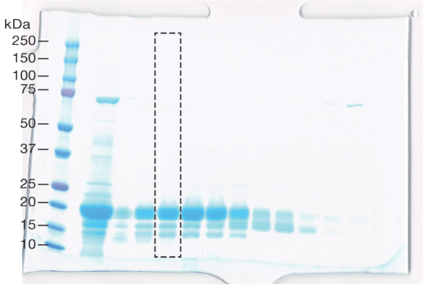

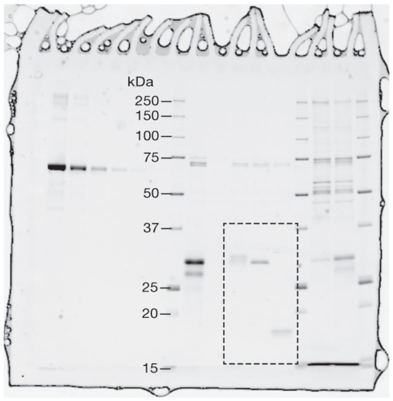

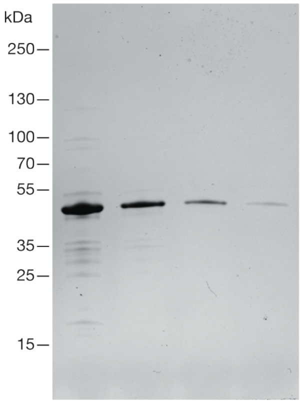

Arif et al., Supplementary Data Fig. 1.

Figure 3b (100 nM target)

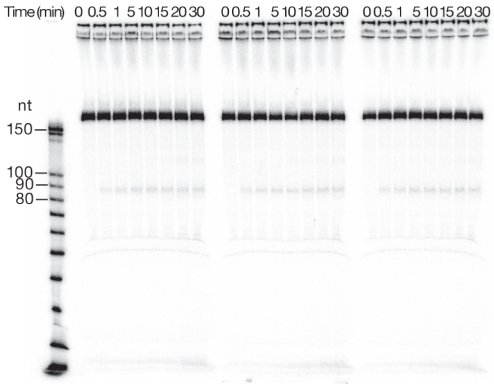

Figure 3c and Extended Data Fig. 5

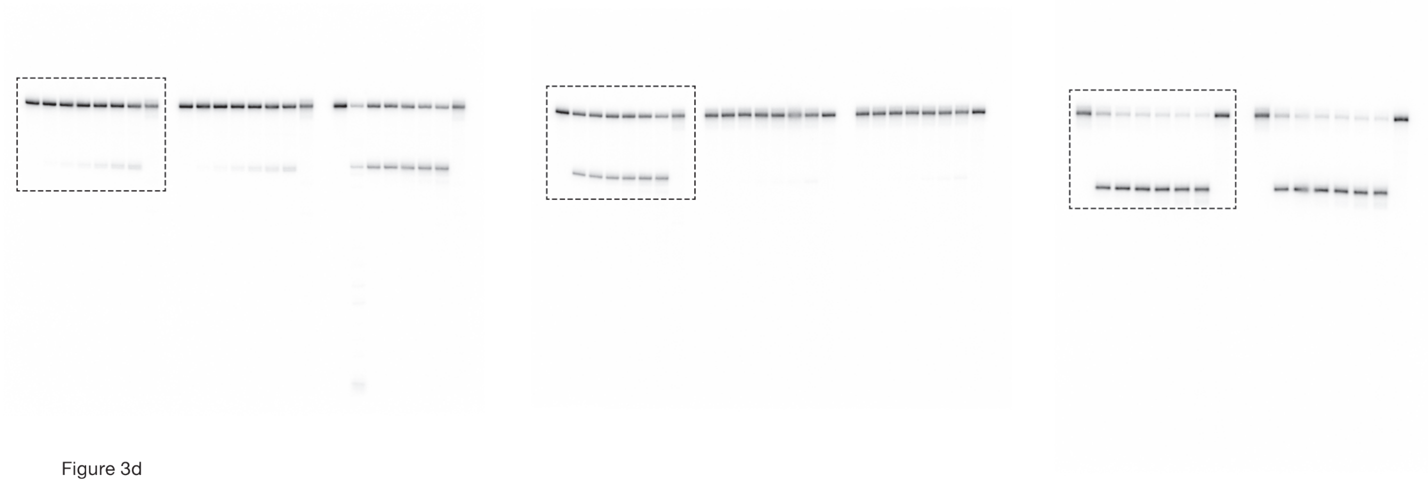

Figure 3d

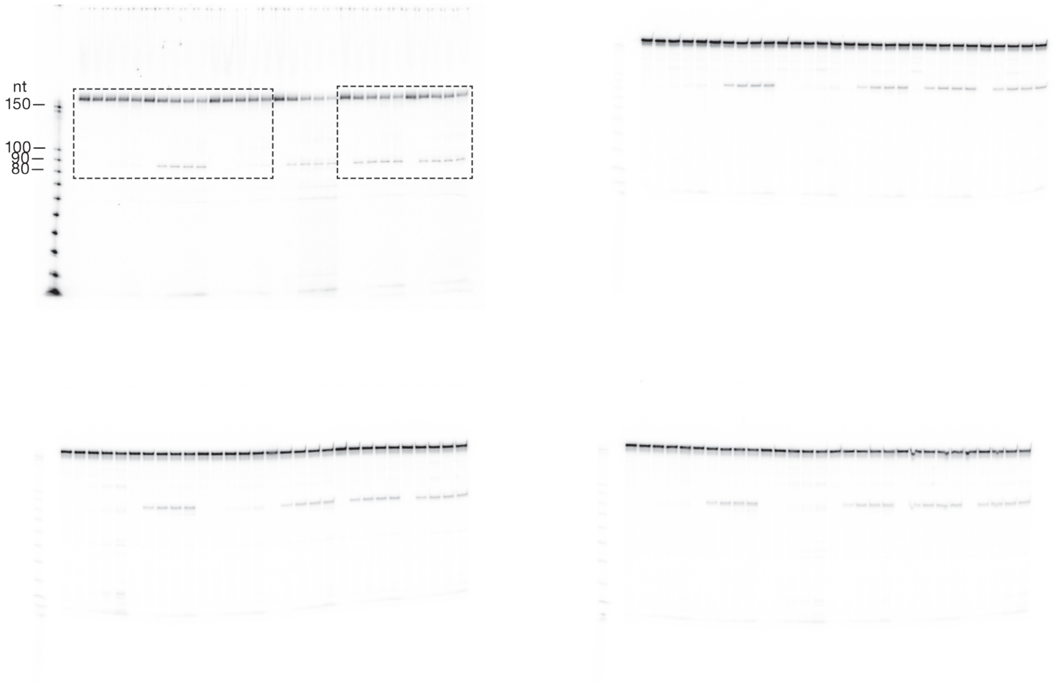

Arif et al., Supplementary Data Fig. 1.

Figure 3e

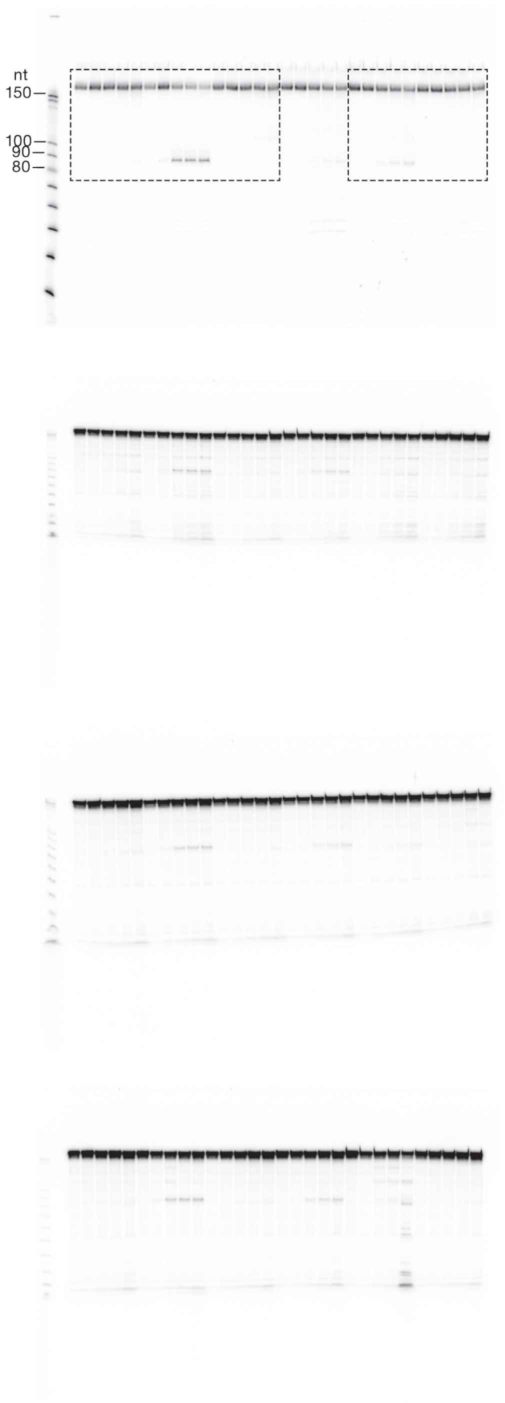

Arif et al., Supplementary Data Fig. 1.

Figure 3g

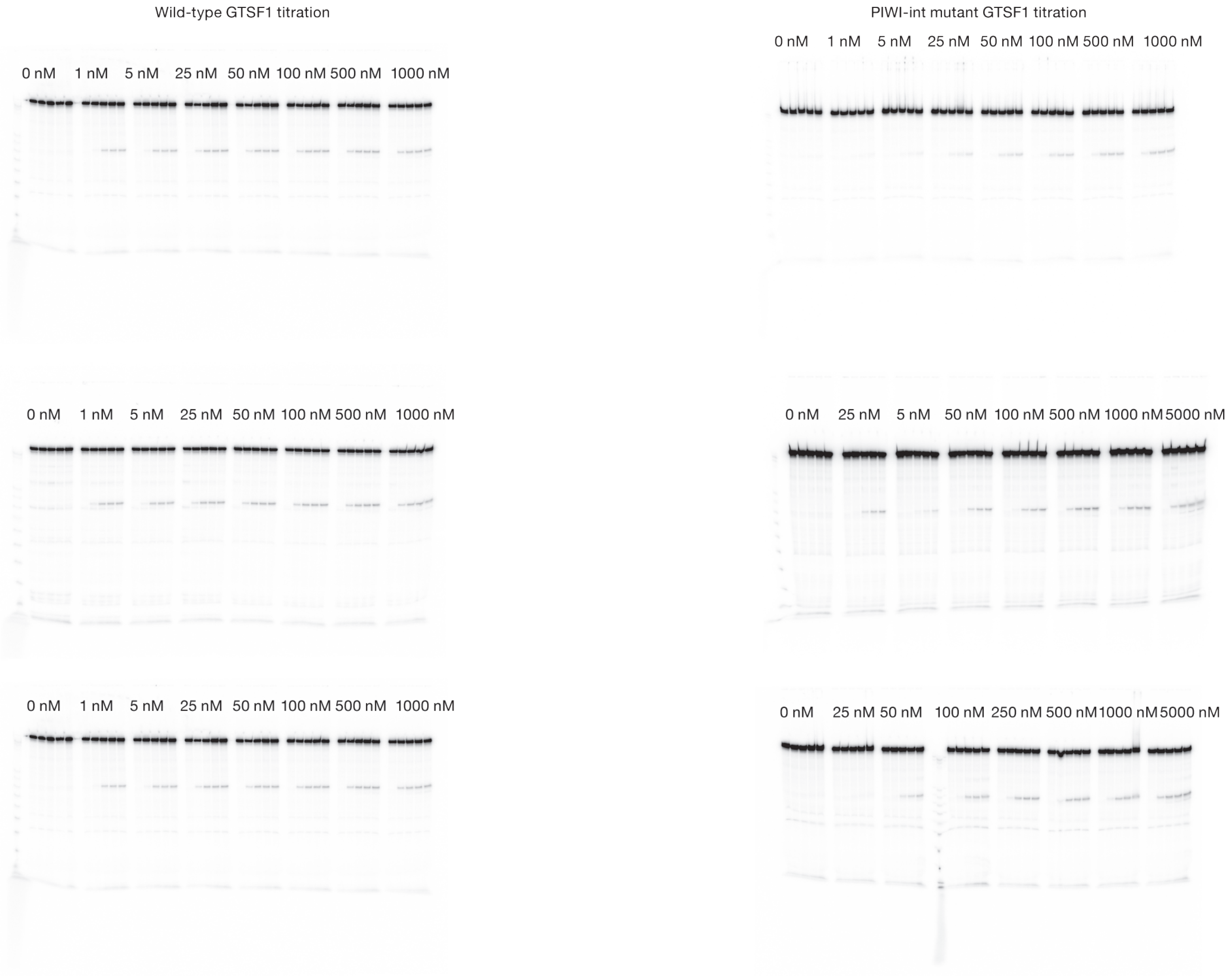

Figure 3h

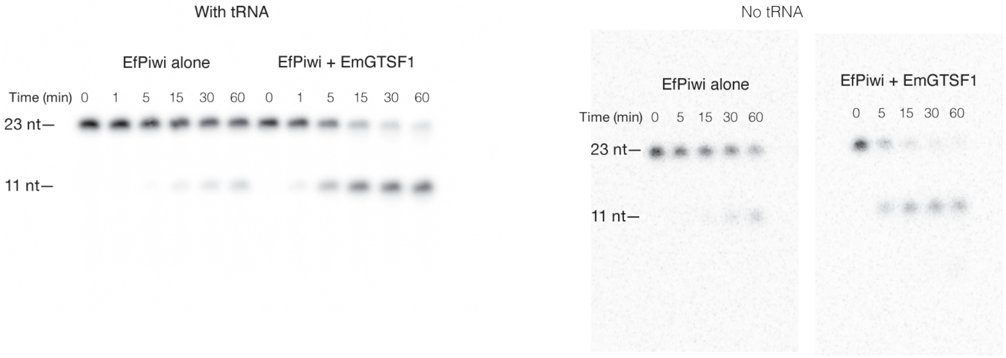

Arif et al., Supplementary Data Fig. 1.

Extended Data Fig. 6a

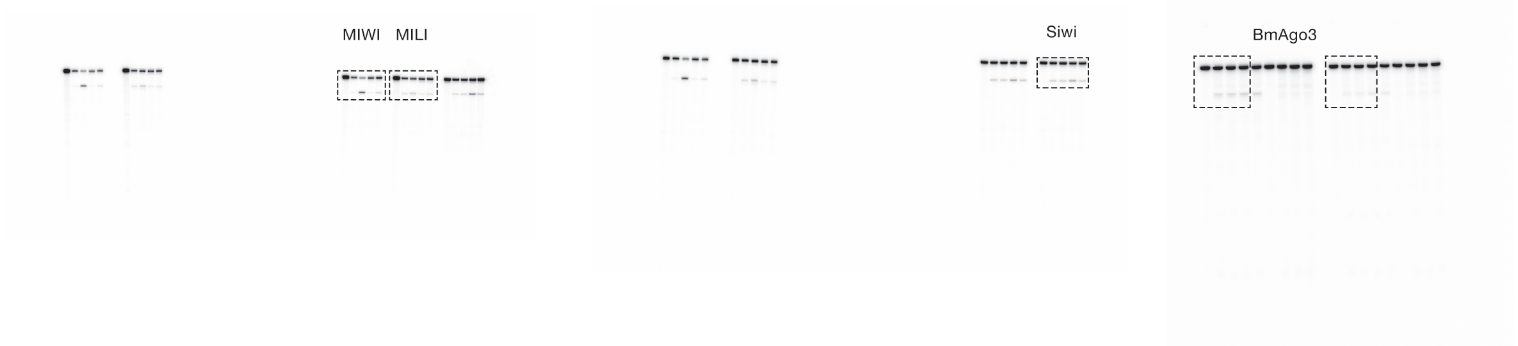

Extended Data Fig. 6b

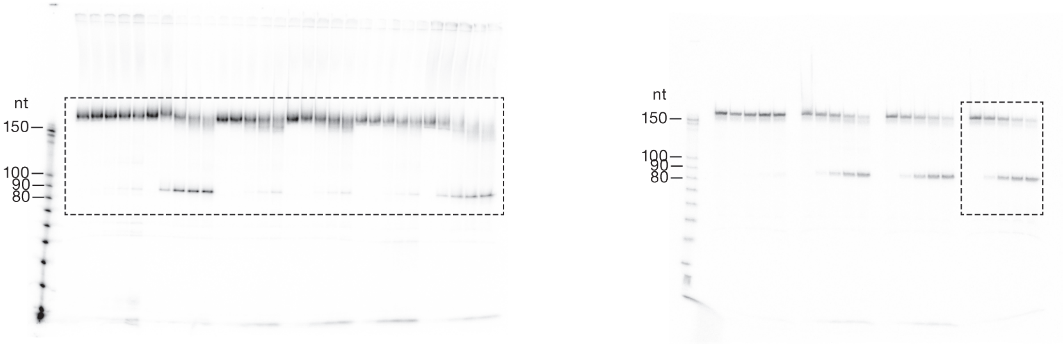

Figure 4a (Top)

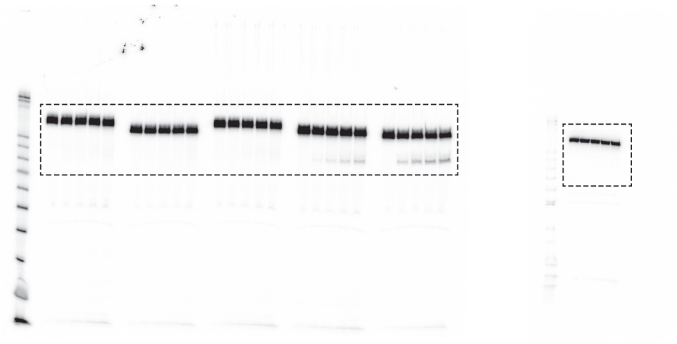

Figure 4a (Bottom)

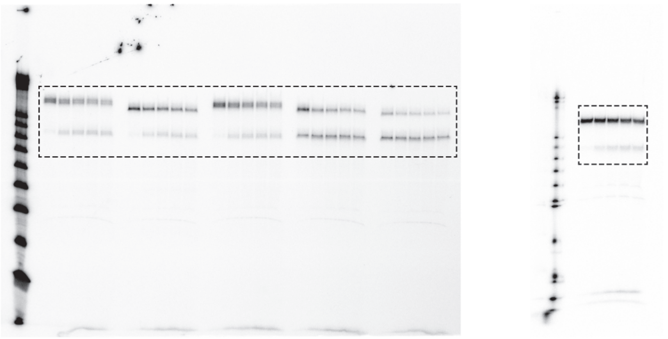

Arif et al., Supplementary Data Fig. 1.

Extended Data Fig. 8a

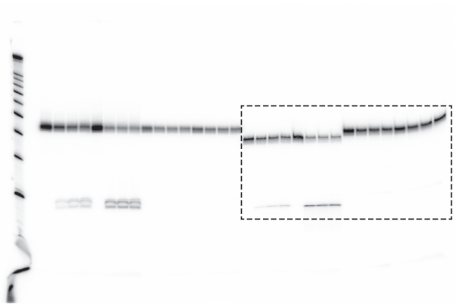

Extended Data Fig. 8b

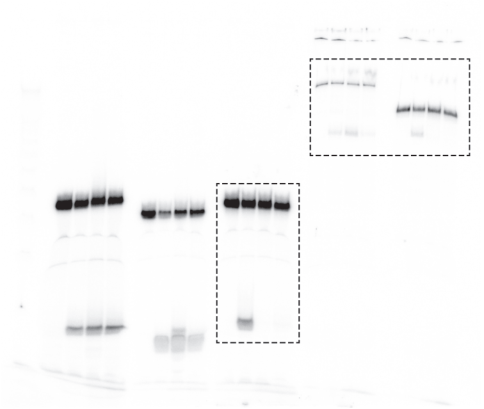

Figure 4b

MIWI (2–30 target) MIWI (2–16 target) MILI (2–30 target) MILI (2–16 target)

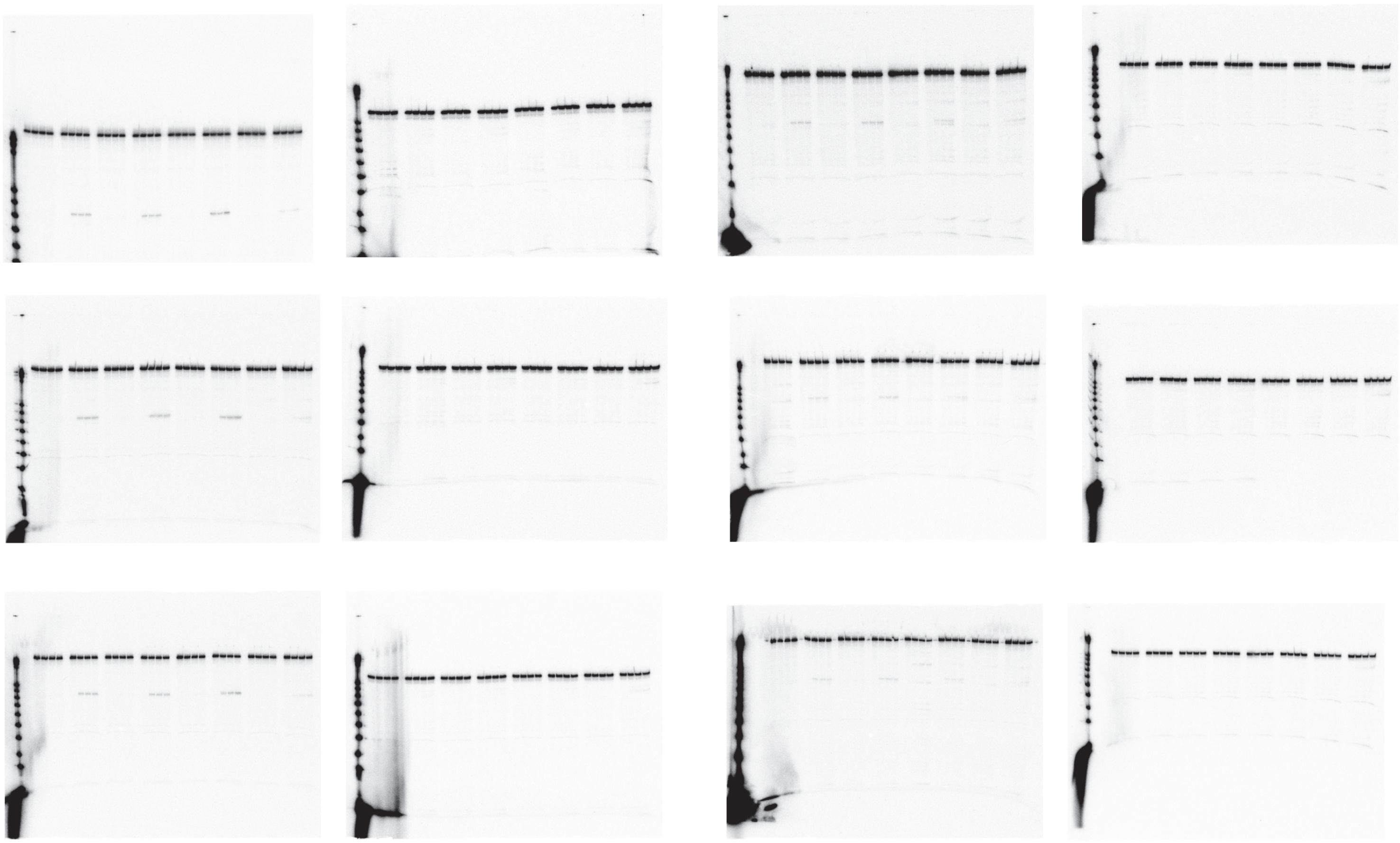

Figure 4c

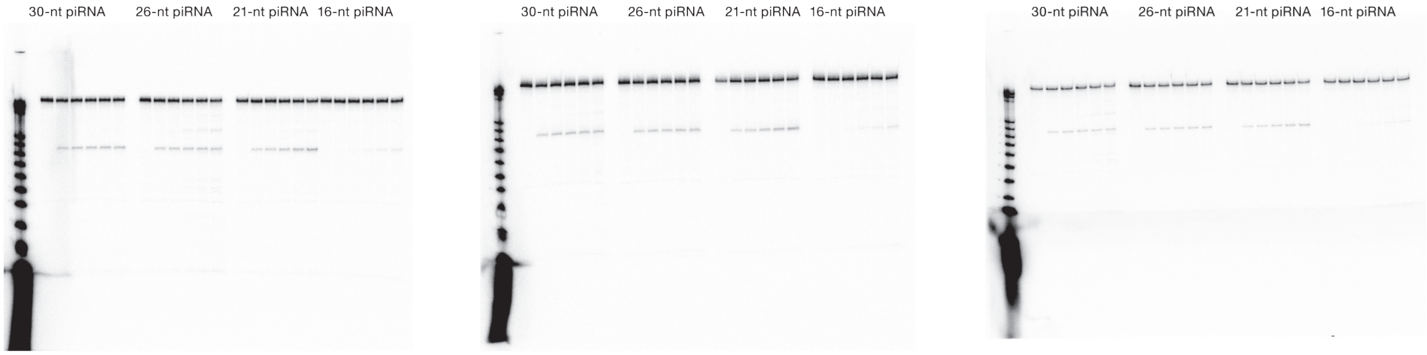

Supplementary Data Fig. 1. Uncropped gel images. Source electrophoretic data used in the indicated main and extended figures. Dashed boxes indicate gel cropping.
