# Supplementary material for: GTSF1 accelerates target RNA cleavage by PIWI-clade Argonaute proteins
Source: Nature. 2022 Jun 30;608(7923):618–25. doi: 10.1038/s41586-022-05009-0 (PMC9385479; doi:10.1038/s41586-022-05009-0)
Supplement: Supplementary file 3 — Individual MIWI kburst graphs using wild-type or PIWI-int mutant GTSF1. [file 41586_2022_5009_MOESM3_ESM.pdf]

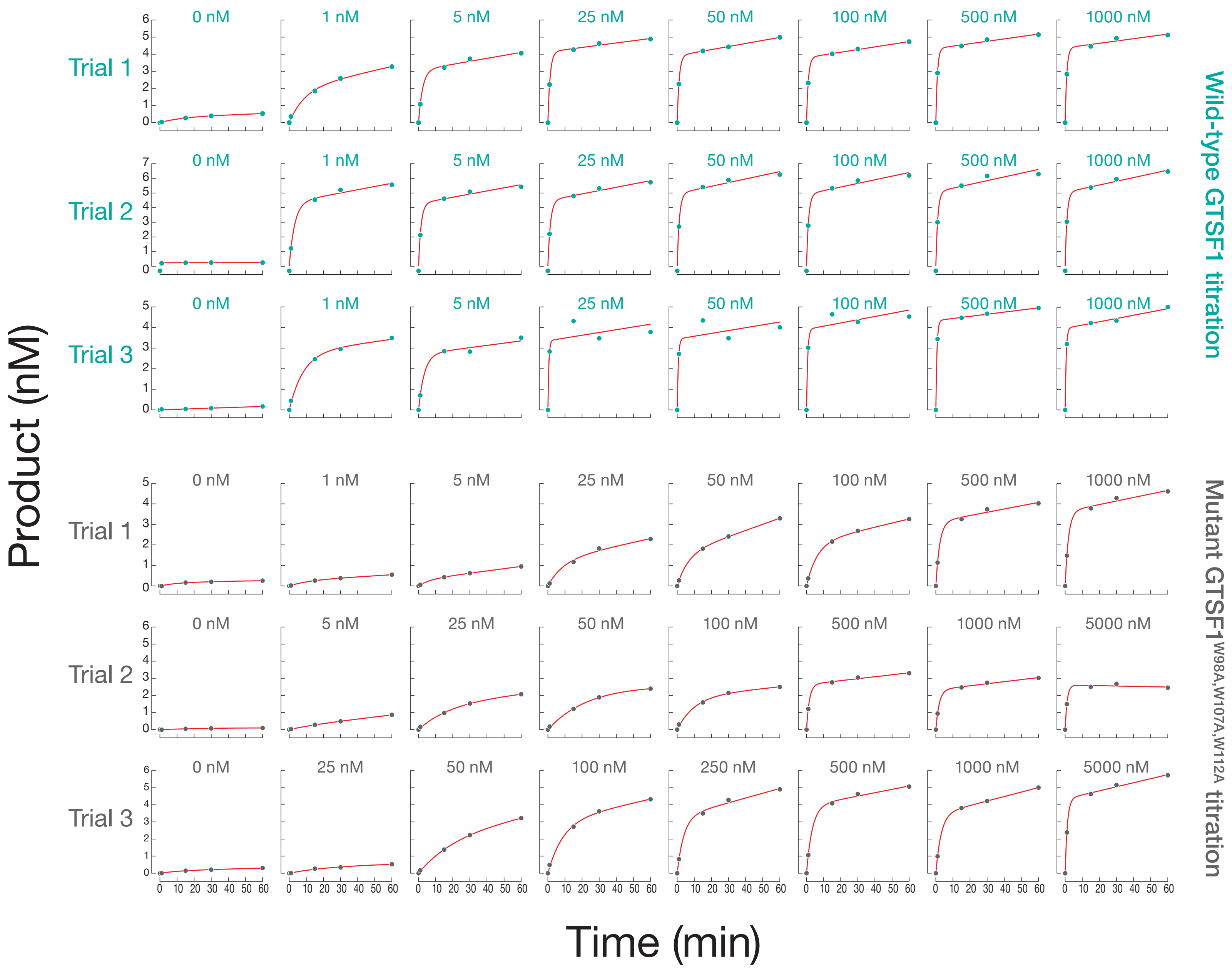

Supplementary Data Fig. 2. Wild-type and PIWI-int mutant GTSF1 titration. Time-course graphs showing effect of titrating wild-type and PIWI-interacting mutant GTSF1 on pre-steady-state catalytic rate of MIWI. Gel source data in Supplementary Data Fig. 1.
