# Supplementary material for: GTSF1 accelerates target RNA cleavage by PIWI-clade Argonaute proteins
Source: Nature. 2022 Jun 30;608(7923):618–25. doi: 10.1038/s41586-022-05009-0 (PMC9385479; doi:10.1038/s41586-022-05009-0)
Supplement: Supplementary file 4 — Oligonucleotides and DNA sequences used in this study. [file 41586_2022_5009_MOESM4_ESM.docx]

| **Supplementary Table 1.** Oligonucleotides and DNA used in this study | | |
| --- | --- | --- |
| **Name** | **Sequence (5′-to-3′)** | **Notes** |
| AGO/PIWI RNA guides (m, 2′-*O*-methyl; phos, 5′ phosphate) | | |
| p30.47 | /phos/UGA GGU AGU AGG UUG UAU AGU AUC CAG AGG | First 21 nucleotides correspond to *let-7* miRNA sequence; 3′,2′ OH; orange label in figures |
| p30.50 | /phos/UGA GGU AGU AGG UUG UAU AGU AUC CAG AGmG |  |
| p30.50DNA | /phos/TGA GGT AGT AGG TTG TAT AGT ATC CAG AGmG | DNA guide |
| p21.46 | /phos/UGA GGU AGU AGG UUG UAU AGU |  |
| p30.53 | /phos/UUA GGU AAC CCA GUA GAU CCA GAG GAA UUmC | Guide sequence targeting luciferase in pGL2 plasmid; red in figures |
| p26.53 | /phos/UUA GGU AAC CCA GUA GAU CCA GAG GmA |  |
| p26.53 (3ʹOH) | /phos/UUA GGU AAC CCA GUA GAU CCA GAG GA |  |
| p21.1175 | /phos/UUA GGU AAC CCA GUA GAU CCA |  |
| p16.53 | /phos/UUA GGU AAC CCA GUA G |  |
| FLAG-Siwi/MIWI/MILI pre-piRNA | /phos/UCG AAG UAU UCC GCG UAC GUU AUG CUA GCU GAU CCU GAC CGC UGA GUC GU |  |
| L1MC piRNA | /phos/UAA CUA AAU ACU AUG CAA GCU GUA GGU CCmU |  |
| Scpep1 piRNA | /phos/UAU CAA AGA AGG UUU UCA GGA GAC UAU GCmA |  |
| AGO passenger strand | | |
| p21.443 | /phos/UAU ACA ACC UAC UAC CUC AUU | PS for 21.46 |
| p21.1176 | /phos/UGG AUC UAC UGG GUU ACC UUC | PS for 21.1175 |
| Target RNA oligos | | |
| 5′ CH_3_O/Tar3 | /5′ CH_3_O/UAU AGA AAA AAU AAC UAU ACA ACC UAC UAC CUC AAU GCU | 2-21 matched target against let-7 type guide sequences |
| 5′ CH_3_O/Tar4 | /5′ CH_3_O/UAU GAA GAG AUC GUA ACC ACA ACC UAC UAC CUC AAU GCU ACA | 2-16 matched target against let-7 type guide sequences |
| Primers for IVT targets | | |
| Pr8F_T7_pGl2 | GCG TAA TAC GAC TCA CTA TAG GGT TTT AAT GAA TAC GAT TT | Forward Primer to add T7 promoter to template |
| Pr9R | CCC ATT TAG GTG ACA CTA TAG ATT TAT ACC TAG TTA AAC AGC GGA ACT GTG TAT AAA AGG TTG AGG TAG TAG GTT GTA TAG TAT CCA GAG GAA TTC ATT ATC AGT G | Reverse primer for fully complementary let-7 target |
| Pr_let7_(2-16)R | GCA CTG TTT TCA TGA AGA CAT GAA GAG ATC TGT AGC ATT GAG GTA GTA GGT TGT GGT TAC GAT CTC TTC ATG AGG AAT TCA TTA TCA GTG | Reverse primer for 2-16 let-7 target |
| Pr_Luc_Tar1R | TCC GGA ATG ATT TGA TTG CC | Reverse primer for fully complementary luciferase target |
| Pr_Luc_tar8R | GCG GAA GGG CCA CAC CCT TAG GTA ACC CAG TAG ATC CAC CTC TGG ATA TTA TCA GTG | Reverse primer for 2-21 match luciferase target |
| Pr_Luc_tar7R | TAT GCG GAA GGG CCA CAC CCT TAG GTA ACC CAG TAG ATC CTC CTC TGG ATA TTA TCA GTG | Reverse primer for 2-20 match luciferase target |
| Pr_Luc_Tar6R | TTC TAT GCG GAA GGG CCA CAC CCT TAG GTA ACC CAG TAG ATC TTC CTC TGG ATA TTA TCA GTG | Reverse primer for 2-19 match luciferase target |
| Pr_Luc_Tar5R | GCG GAA GGG CCA CAC CCT TAG GTA ACC CAG TAG ATA TTC CTC TGG ATA TTA TCA GTG | Reverse primer for 2-18 match luciferase target |
| Pr_Luc_Tar4R | TTC TAT GCG GAA GGG CCA CAC CCT TAG GTA ACC CAG TAG AAA TTC CTC TGG ATA TTA TCA GTG | Reverse primer for 2-17 match luciferase target |
| Pr_Luc_tar3R | TAT GCG GAA GGG CCA CAC CCT TAG GTA ACC CAG TAG GAA TTC CTC TGG ATA TTA TCA GTG | Reverse primer for 2-16 match luciferase target |
| L1MC_57_R | ACA CTA TAG ATT TAT ACC TAG TTA AAC AGC GGA ACT GTG TAT AAA AGG TTA ACT AAA TAC TAT GCA AGC TGT AGG TCC TAT AGG TCT CCA ATT CAT TAT CAG TGC AAT | Reverse primer for L1MC target |
| Scpep1_59_R | ACA CTA TAG ATT TAT ACC TAG TTA AAC AGC GGA ACT GTG TAT AAA AGG TAA TCA AAG AAG GAT TTC AGG AGA ACC ATC AAT AGG TCT CCA ATT CAT TAT CAG TGC AAT | Reverse primer for Scpep1 target |
| Sequence-affinity purification (m, 2′-*O*-methyl) | | |
| luciferase capture oligo | /Biotin/mAmCmU mAmCmU mGmCmA mGmCmA mCmAmA mCmCmC mUmAmC mCmAmA mAmUmU mAmCmC mUmAmA mCmUmG mC |  |
| luciferase competitor oligo | GCA GTT AGG TAA TTT GGT AGG GTT GTG CTG CAG TAG T | DNA oligo |
| let-7 capture oligo | /Biotin/mAmUmA mGmAmC mUmGmC mGmAmC mAmAmU mAmGmC mCmUmA mCmCmU mCmCmG mAmAmC mG |  |
| let-7 competitor oligo | CGT TCG GAG GTA GGC TAT TGT CGC AGT CTA T | DNA oligo |
| Primers for BmAgo3 targets | | |
| Target-F | GCG TAA TAC GAC TCA CTA TAG TCA CAT CTC ATC TAC CTC C | Forward Primer to add T7 promoter to the template |
| Target #1 (pi848-perfect target-R) | AGG TGA CAC TAT AGA TTT ACA TCG CGT TGA GTG TAG AAC GGT TGT ATA AAA GGT CGA GTG ACA TGT TTG GAA GCA ACA GCA TCC AGA GGA GTT CAT G | Reverse primer for fully complementary target |
| Target #2 (pi2148ds-perfect target-R) | AGG TGA CAC TAT AGA TTT ACA TCG CGT TGA GTG TAG AAC GGT TGT ATA AAA GGT AGG GCC AAG ACC AAA TAT AGT ACG TTT CAT CCA GAG GAG TTC ATG | Reverse primer for fully complementary target |

| Construction of *Gtsf1^KI/KI^* mouse | | |
| --- | --- | --- |
| HDR donor | ACTTTGTCCAATTCACCGACAGCCTGTTATTGGGGGAAAGGAATCAAATCACCTGTTAACAATTTGATGGGGAAGATAAGAGACAGCTGAATGATGGTGTTATGAGTTCCAGCGTCCTTGGCCTACTTGGAATCAGTCTGTTTGCTAAGAAAGAATAGGTCAGCTCTCTAAGTCGCTGAATATCTTTACTCATCTTTTTCAAAATAATAATAATAATGTAATTCTTGGTTTTTCCTTAAAACCTTTTTTTATTTTTAACAAAGTATGACATAACTGTAGTAGAAGTTCCATACGGGTTAAGATAGTTTAACTAATTCTCAGATAGTTTCCCCAATGATTGCCATTAACAAATGGGCTCTGGAGCAAAGTCCGAAACTAGAGTTTAGTTGATCACCCCTATTGCTTAACAAAGGTTCTAATGCTGCCTATGTCATAGAATGGGGGGGAAGGGGGTGTGGAATTTTTGTGAGAGGCCAAAGGAGAGCTTACCTAGAGACAACAGCTGTATGTGCATCCAATTTTCAGAAGATTCCTATGGGAGAGGTTATATACAAAATTAGTTTTCCCATAATACGTGAACTAAATCAGTGTTCAATAGAGATTCCAGTAAAAGAGAATTCAAACTCAGAGACATTTCCTCTTTTAATTTTTTTCATGTTTGTTATATTTGTTCTCTTAGATGTTCTCTATGAGCTCTTAATTTTTTTAACATTCACATATATTCCCTCAGTGCAGTTAAGTAATCAGTACTGACTAAAACAGTATTTATCTTGTTATTATATAGAAATGAGAGGAACAAAATGAAAAATGGCTAGAAAGAGAGAATATTGGTGTGACGTTTTCTGATCATGATTAAGAAAAGTTACTAAGCTGGGTATGGTTGAAAATGCCTTTAATCCCAGAACTCAGGAGGCAGAGGCAGGTGGATCTCTGTGTGTTCAAGGTCAGTCTGGTCTGCATATTGAGTTCCAGGACAGCCAAAACTATACAGAGAAACCTTGTCTCTTAAAAAAGAAGAAAAAGAAAGGTCATTAAATGAGATGTAGCTTTTATTTTACATGTGTTTGTGTGCATTTTGCCCACAGAAGCCAGAAGAGGGCATCTGATCCCCTGAAACTGGAGTTATAGGCAGTTGTAAGCCATGTGGGTGCTGAGAATCAAACCCAGATGTCACTAAGAACACCCAGAGCTCTTAACCATTTAATCATCTTTCCATCTGGAAAGAAGATCTTTAAAGTAGTTTATCCAGAAGTCTAAATACTGGTATTTTTATGACCTTGACTTGGCCCCCATTACTTATGTTAAATACCATAACCCCTAATAGCATGTAAACAGTGGCAGGTGTTGTAATACCACTTAAAATAAATTGTTATTATTGTCACCTGAATGTAGTTTGTCAGTTGTCATATTTGACCTAGCAATTTATTCCAAGAATGTGAACTATGCAGTGATGCTAAACTTAGTCATAGGAGTAAAGAATGCCTTTAAGAAGAAACTGGAGAGAAGGTTGTATGCTAAGGGTTTATTTTATTGAACTTGGGGCAGCTTCAGGTCCAGGAATGACTCCACATCTTAGGGCTACTATTTGATGCGTTTCATTTCTGATTCTCTGAACCATGCTTTTTCCTCTAGCACTTGAACAGGACTCCTTTGCCATCAGC**ATG**GACTACAAGGACCACGACGGTGACTACAAGGACCACGACATCGACTACAAGGACGACGACGACAAGGGCTCCGGATACCCATACGATGTGCCAGATTACGCTGGATCAGGCGAAGACACTTACAGTATGTACTACTTGCTGCTTTTAGAAAATAATACAATATTTATGTGTAGTTTGATGCCTGACTAGACTGTATGTTGTGAAAACTTCAGATTCCTCCCTGAAATGCTGAGAAAGAAAGGTCTGTGTGATATTGAGGTATACAGATGCTCAAATTTTAAATAAGTAACTTTAGTCTACAGCCATATTACCCTGAAAAAAAATGGTCTGAAACAAGCACTTTAAACTTAAGTAATTAGACATATGAGAACCAACTGTAATGATACACAACTGTAATCCCAACTCATAGGAAGCTGAATCATGGGGATCACAACTTCTAGGCCATCATGGGCTTCACAGAAAGACCATCAAAAGATAAACCAGTAGTCCACTGAGATGGCTCAAGGGTGAAGTGCTTATTACCATAAAAGCCTGACAACCTTAGTTTGAATTTTAGAACTCTTGTAAAAGTAGGAAAGAAAACTGAATCTATAAAGTCGTCCTCTGACCTACACACAGACCACACACAATAATAAGTACAAAATTTTTACAATACTGGGGATGTAACTCAGTGGTAGAGCATTTGTGTAGCATTCTCAAGACCCATATTTTAATACCAAGATAATAGGTAGAGAGAGAGAGAGAGATATGAATGATTGATTGGTGGTTAAATTGGTGACCAAGATAGATGGATGAATGGATGATTGATAAGTAGATGGATGGGTGGATGGATGTAGATAAATAGATGGATCATTGATAGATAGATAGATAGACAGATTCTTTTCAAGGTAGAACAGTAAGTTAGAGCTGACCTAAATTGCCAGACTAAGTAAAGCATTTATGGTAGTTCTCATTATCAACTTTGACACATGCTCTATATGAGCAGTTACAATATGTGTGAATCATGGGGTACCTCTGTTCTGTCAACTGCTGACATTTGCTAGTGATAGAATGGATTAAATGAAGGAAAACTAATAAGAATTTCATCTGTGACTGCTCCCTGGGTGTTCTGAAGCTTCAGAAATAAGAAGAGATTGCTGGCTAAGAGCTCAGTGGCACAGTAGCATGCCTGTCCTCTGGGGCCTTGCCTCCCTGTTCATTCTGTCCTTCAGATCACCTTTCAAGCTGATTTGCATCATGACCGTGGTTAGCAATTTATTCCATGTTTGCCACTCTTCCAGCGAAACTGTCAGTTTCAATTCCCCTCTGGTTTCTGATTTTCATTGCAGTCTCCTGGTTTATAAGCTTCTTATCAATGGTGAATTGTTTGTCTTGCTTTTATCCTTACCTTCTGGGGTGGCAAGAGTGTTTTATTAGGTGCCTTTTGCTCTCAGAATGCACTATTTAACTTTCTTTCCTTAACCAGGCTTGTTAAGATTTGGGGCATAGCCTGAGGGCAGCTTTGAATAACATGGTATAGGTTCTGGGTTTTCTTGACACAGCAGTCTGTGTCTACAATTGTCTTAGCTGTAGGAGGTTATTTTTTATTTTAGCTGGCCAGTTTGGTAGTCCTTAGATTAAGGAACTTTTTTCTCATCTATGTTCCAAAACATGAAAAGTAAAATCACCTACCTGACAAAAGTAATGAGATCATTGTCAGGGGAACGAGAGCAGCCAATACAGCATACAGGAAGGAGTAAAGCAAAGAACAAATTGGGGGGAAACTCTAGTTATTAATACTTCATAGAACATTCAATTGAAGCTATCAACTTCTTAACTAGAAATAGACAAGGATGCTAATGAGATTATTCTCAACTACGGCTAGCTAGATTGAGAGAGTGTTGGAAAGTGCAGTGGGC | Left and right homology arms; **ATG**: *Gtsf1* start codon; 3XFLAG-HA knock-in cassette |
| sgRNA target sequence | GTCTTCCATGCTGATGGCAAAGG | PAM sequence |
| Primer F1 | CTGTTTTACTGAGACCTGCAACTTT | Genotyping and validation primers |
| Primer R1 | GATCCAGCGTAATCTGGCACAT |  |
| Primer F2 | ACTACAAGGACCACGACGGTG |  |
| Primer R2 | GACTGGATTGCTGGTGTAACAATA |  |
